# Variation characteristics of different plant functional groups in alpine desert steppe of the Altun Mountains, northern Qinghai-Tibet Plateau

**DOI:** 10.3389/fpls.2022.961692

**Published:** 2022-09-13

**Authors:** Ailin Zhang, Xiangyi Li, Fanjiang Zeng, Yong Jiang, Ruzhen Wang

**Affiliations:** ^1^State Key Laboratory of Desert and Oasis Ecology, Xinjiang Institute of Ecology and Geography, Chinese Academy of Sciences, Ürümqi, China; ^2^Xinjiang Key Laboratory of Desert Plant Roots Ecology and Vegetation Restoration, Ürümqi, China; ^3^Cele National Station of Observation and Research for Desert Grassland Ecosystems, Cele, China; ^4^University of Chinese Academy of Sciences, Beijing, China; ^5^School of Life Sciences, Hebei University, Baoding, China

**Keywords:** plant functional groups, Qinghai-Tibetan Plateau, grassland ecosystem, species diversity, C, N, P

## Abstract

In grassland ecosystems, the plant functional group (PFG) is an important bridge connecting individual plants to the community system. The grassland ecosystem is the main ecosystem type on the Qinghai-Tibet Plateau. Altun Mountain is located in the key grassland transcontinental belt of the northern Qinghai-Tibet Plateau. The composition and changes in the PFG in this ecosystem reflect the community characteristics in the arid and semi-arid extreme climate regions of the Plateau. The main PFGs were forbs and grasses, and the importance values (IVs) accounted for more than 50%. Plant species diversity of the community was influenced by the IV of the legumes, and the increase in legumes would promote the increase in plant community diversity. The C, N, and P contents of plant communities were mainly influenced by forbs and grasses, and the relationship between forbs and C, N, and P was opposite to that of grasses. However, under the influence of different hydrothermal conditions, forbs and grasses as dominant functional groups had a stronger correlation with community and soil nutrients. This indicates that the dominant PFGs (forbs and grasses) can dominate the C, N, and P contents of the community and soil, and legumes affect community composition and succession. In this study, we analyzed the changing characteristics of functional groups in dry and cold extreme environments and the difference in their impacts on community development compared with other grassland ecosystem functional groups.

## Introduction

The grassland ecosystem is an important terrestrial ecosystem, and it is an important transitional zone in the arid and semi-arid regions (Dong et al., 2010). With the intensification of global climate change an–d human activities, the community composition of the grassland ecosystem has changed significantly (Li et al., 2000). To better summarize and analyze the vegetation changes in grassland communities, plants with similar attributes are classified into groups called plant functional groups (PFGs) (Solbrig, 1993). PFG connects individual plant traits and community ecological processes and is an important method for simplifying community-level processes (Eviner and Chapin, 2003). PGFs act as an assemblage of similar species that can connect ecosystems and species, establish a direct relationship between vegetation and climate, and make comparisons at different regional scales (Skarpe, 1996; Lavorel et al., 1997).

Previous studies have shown that the change in some dominant species of a community will have a great impact on the plant community composition (Hooper and Vitousek, 1997). The effects of PFGs on community structure and function are mainly due to the ecological strategies of different species in response to environmental changes. Species richness can alter ecosystem functions, and different functional groups have different effects on species richness in many studies (Cardinale et al., 2006; Fischer et al., 2015). In addition, PFG plays a key role in determining the impact of species loss on ecosystems (Mclaren and Turkington, 2010). In addition, studies have shown that the increase in soil nitrogen (N) content and the high content of soil phosphorus (P) will decrease the community diversity (Baer et al., 2003; Kattge et al., 2011) and affect the composition of PFGs by changing the dominant functional group (Li et al., 2020). Soil nutrients not only affect the composition of PFGs but also receive negative feedback from the change in PFGs. The effects of different functional groups on soil nutrients were mainly caused by altering litter quantity, amount of root exudates, and intensity of resource utilization (Porazinska et al., 2003). The changes in PFGs may modulate the response of soil carbon (C) to long-term global climate change (Du et al., 2018).

In extreme cold or dry environments, the composition and ecological strategies of PFG for coping with the extreme environment differ from those of other ecosystems with mild environmental conditions. A study has pointed out that herbs invest preferentially in structures for persistence (K-strategy) in the alpine grasslands (Patty et al., 2010). Therefore, different PFGs have contrasting adaptations in alpine or arid environments. Studies have shown that with the degradation of alpine grasslands, the density per unit area of vascular plants, grasses, and sedges decreased on the Qinghai-Tibet Plateau (Yang et al., 2013). In arid environments, the biomass allocation of PFGs was different in humid and subhumid areas in response to the stress caused by the arid environment (Carlsson et al., 2017). Drought would decrease the PFG of legumes and increase the functional group of grasses (Stampfli et al., 2018). At the same time, changes in PFGs can also affect the environment by modifying the water cycle (Wu et al., 2019). For example, due to the difference in root biomass, leguminous functional groups can increase the surface soil water content, while grass functional groups can decrease the surface soil water content (Ravenek et al., 2014). But in cold environments at high altitudes, PFGs are more likely to be affected by soil nutrients (Dormann and Woodin, 2002).

The alpine grassland is the main vegetation type on the Qinghai-Tibet Plateau, which not only maintains the plateau ecosystem and production but also has an important impact on the ecological security of the downstream region (Cao et al., 2004). The Altyn Tagh lies to the north of the Qinghai-Tibet Plateau, and the natural climate features are cold and dry, belonging to the Qinghai-Tibet Plateau cold climate. The first alpine desert nature reserve was established here in the world, and it plays an important role in the protection of alpine desert ecosystems and their unique species (Sha et al., 2016). The vegetation types are different from those of other grassland ecosystems in the Altun Mountains. At the low altitude, the alpine desert vegetation is the main vegetation type, and at the high altitude, the vegetation types are composed of alpine desert and alpine grassland (Sha et al., 2016). Therefore, community composition and functional groups are more abundant at higher altitudes than at lower altitudes. In the extreme environment at a high altitude in the Altun Mountains in the northern part of the Qinghai-Tibet Plateau, what are the adaptive characteristics of PFGs to this environment? Therefore, the aims of this study were as follows: (1) as in typical grassland ecosystems, legume functional group affects community diversity; (2) forb plants are more adaptable to extreme environments than other functional groups due to their rapid adaptation to the environment.

## Methods and materials

### Study site and experimental design

Altunshan Nature Reserve is located in the south of Altyn Tagh and the north of the Qinghai-Tibet Plateau (87°10'E−91°18'E, 36°N−37°49'N). The study area belongs to the plateau climate, and the climate is dry and cold. The mean annual temperature is approximately 0°C, annual rainfall is approximately 300 mm, and the sunshine intensity is high. The soils are mainly developed from alpine desert soil, alpine steppe soil, and high mountain desert soil, and some areas also have alpine meadow soil. The reserve is rich in vegetation types, which mainly include deserts, grasslands, and marsh. The height of the grass layer is mostly 5–20 cm, the coverage is 10–30%, and the coverage can reach 60–80% in some alpine grassland. The dominant species include *Stipa purpurea* and *Kobresia robusta*, and common companion species are *Carex kunlumsannsis, Koeleria cristata*, and *Oxytropis falcata* (Wu Y. et al., 2013).

**Figure 1:**
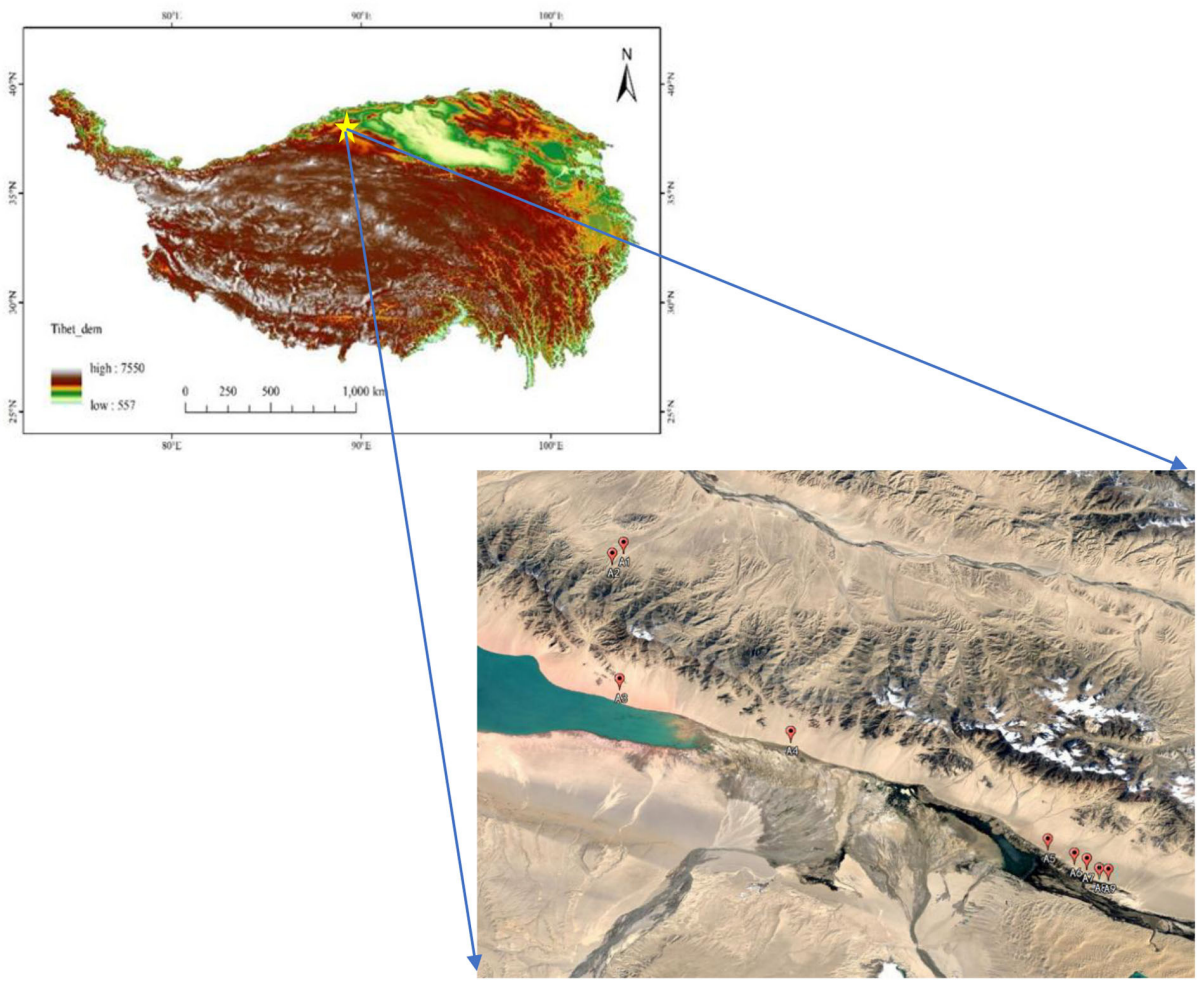
Distribution of sample points.

In August 2019, we carried out the field survey, and a total of 9 representative plots were randomly selected in the Altunshan Natural Reserve. The 9 representative plots belong to the same vegetation type. The altitude of all the samples was higher than 3,700 m (A 1—3,720 m, A 2—3,790 m, A 3—3,740 m, A 4—3,720 m, A 5—3,715 m, A 6—3,730 m, A 7—3,740 m, A 8—3,745 m, and A 9—3,790 m), and the vegetation type belonged to alpine grassland (Figure 1). Each plot records its geographical location, including latitude, longitude, altitude, and slope. A total of 5 replicates (100 cm × 100 cm) were arranged in each plot, and the coverage frequency and height of herbaceous plant species in each quadrat were investigated and recorded. The cover of different functional groups and the community were summed up by the per species coverage. From the estimates of canopy coverage per species, we determined the relative coverage of each and entire community (i.e., percent cover). In addition, the dominance of plant species was calculated. Then, all plants were cut from the ground in every quadrat, their fresh weight was recorded, and their weight was called after drying. On completion of the aboveground sample collection, soil samples were collected using the five-point sampling method from 0 to 40 cm with 7 cm drills in every quadrate. Additionally, plant roots were isolated from the soil samples, and their weight was called after drying. According to plant traits, all herbaceous plant samples were divided into four PFGs, namely, forbs, grasses, legumes, and sedges (Supplementary Table A1).

### Soil and plant laboratory measurements

After completing a field survey and sampling, plant samples (aboveground and root samples) were placed in the oven at 105°C for 30 min, then oven-dried at 75°C for 48 h, and the biomass was weighed thereafter. All soil samples were measured at sampling conditions (wet weight) and soon after oven-dried for 48 h at 10°C and then weighed again. Soil pH was determined using a conductometer (1:1 soil-water suspension) and acidimeter (1:5 soil-water suspension). Soil organic carbon (SOC) was determined using the K_2_Cr_2_O_7_ oxidation. Soil total nitrogen (TN) was determined using the Vario Macro Cube—Elemental Analyzer. In addition, soil total phosphorus (TP) concentrations were determined using H_2_SO_4_-HClO_4_ digestion methods. The plant samples were determined for SOC, TN, and TP after drying for 48 h at 75°C, and the determination method is the same as that of soil samples.

### Data processing

#### Importance values

The importance value (IV) of a species can be regarded as the dominance of each species in the community, reflecting the influence of that species in the community (Whittaker and Niering, 1965). The IVs of different vegetation types are obtained through different calculation methods (Qian et al., 2008). The IV of PFGs is composed of the IV of the species that make up the PFG.

The IV was calculated as follows:


$$\begin{array}{l} \text{IV}=\left(\text{C}+\text{H}+\text{F}\right)/3 \end{array}$$

where C denotes the relative coverage, this is given by the coverage of a species in the sample plot divided by the coverage of all species in that same plot; the H denotes the relative height, being the height of a species in the sample plot divided by the height of all species in that same plot; and the F denotes the relative frequency, being the frequency of a species in the sample plot divided by the frequency of all species (Yang et al., 2013).

#### α-diversity indexes

To reflect changes in the composition of different communities, well-established diversity indexes were used in this study to represent species richness, evenness, dominance, community structure, and spatial heterogeneity (Whittaker and Niering, 1965; Zhang et al., 2018). We calculated them based on the species data obtained from the vegetation survey; the four specific indexes derived were as follows:

(1) Patrick richness index (*R*)
  *R* = S
(2) Shannon-Wiener index (*H*′)
  *H'* = −Σ P_i_lnP_i_
(3) Simpson index (*D*)
  *D* = 1−Σ P${}_{\text{i}}^{2}$
(4) Pielou evenness index (*Jsw*)
  *Jsw* = H/lnS

In these equations, *S* denotes the total number of species in a plot; *Pi* indicates the relative IV of the ith species; *S'* is the average species number of the quadrat of the plot; *H*′ represents the number of existing species in the plot and the relative abundance of each species; *D* is the dominance of a given species in the community. *Jws* represents the distribution of all species within the community (Hill, 1973).

#### Data analysis

One-way ANOVAs and the least significant difference (LSD) test were used to analyze the differences in diversity of species (*R, H', D*, and *Jsw*), followed by Duncan's test. Pearson's correlation analysis was used to test the relationship of PFG importance values, biomass (aboveground biomass and root biomass), diversity (*R, H', D*, and *Jsw*), plant nutrients (SOC, TN, and TP), and physical and chemical properties of soil (SOC, TN, TP, pH, and soil moisture).

In the alpine grassland ecosystem, the changes in PFGs are affected by several environmental variables. The multiple linear regression (MLR) model is a method for modeling the linear relationship between the predicted variable and more predictors by fitting a linear equation to the model (Bashir et al., 2019). Therefore, we carried out MLR model analysis on different PFGs and nutrients, biomass, diversity, and other factors in the community as follows:

(5) *Y* = T + T_1_*X*_1_+ T_2_*X*_2_+ T_3_*X*_3_+…

In these equations, *Y* is the value for the parameters of the PFGs, and Tn is the value for each corresponding environmental variable (Kutner and Nachts, 2005). All statistical analyses were implemented using SPSS 22.0, Origin 9.3, and R 3.6.2.

## Results

### PFG importance values of different communities

**Figure 2:**
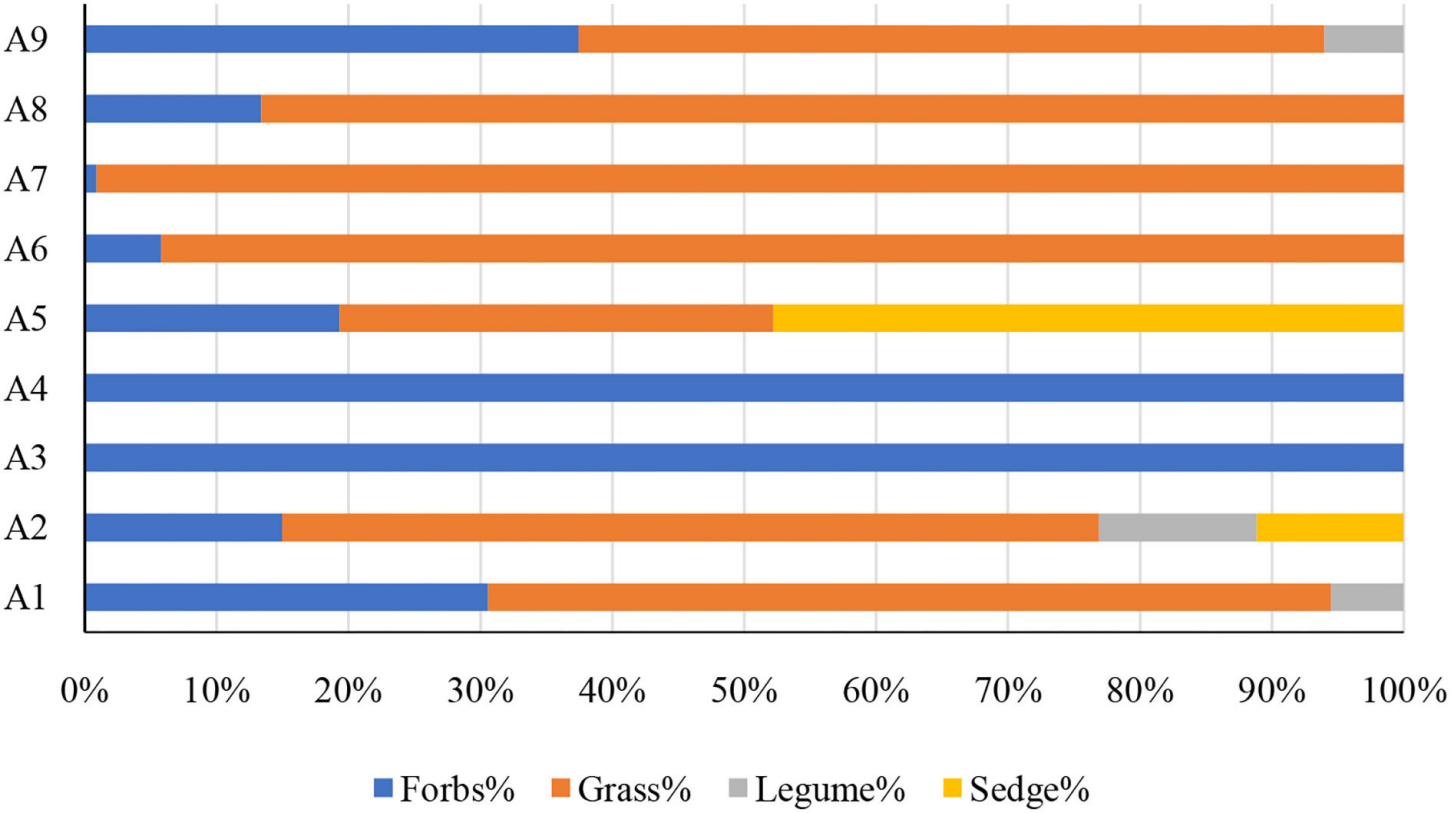
Percentage of important values of different plant functional groups (PFGs).

A total of 22 species were identified from all the sample sites, which were divided into four PFGs according to their traits, including forbs, grasses, legumes, and sedges. The community of the ecosystem is mainly composed of forbs and grasses, and forbs and grasses accounted for more than 50% of the community importance values in all the samples investigated (Figure 2). The percentage of important values of legumes and sedges was relatively low and only existed in a few sample sites (A 1, A 2, A 5, A 9) (Figure 2). In the correlation analysis with biomass, there was a significant positive correlation between forbs with aboveground and underground biomass (*p* < 0.05) and an extremely significant positive correlation with total biomass (*p* < 0.001) (Table 1). There was a significant negative correlation between grasses with underground biomass (*p* < 0.001) (Table 1). But there was no significant correlation between legumes and sedges with community biomass.

**Table 1:** Correlation analysis of plant functional group (PFG) importance values and biomass.

	**Forbs**	**Grass**	**Legume**	**Sedge**
Above-ground biomass (g)	0.342*	−0.218	−0.140	−0.054
Under-ground biomass (g)	0.368*	−0.401**	−0.153	−0.001
Total biomass (g)	0.386**	−0.279	−0.158	−0.050
Under/above biomass	−0.253	−0.081	−0.030	−0.028

The * indicates a significant correlation (*p* < 0.05), and the ** indicates extremely significant correlation (*p* < 0.001).

### Correlation analysis of PFG and community diversity

**Figure 3:**
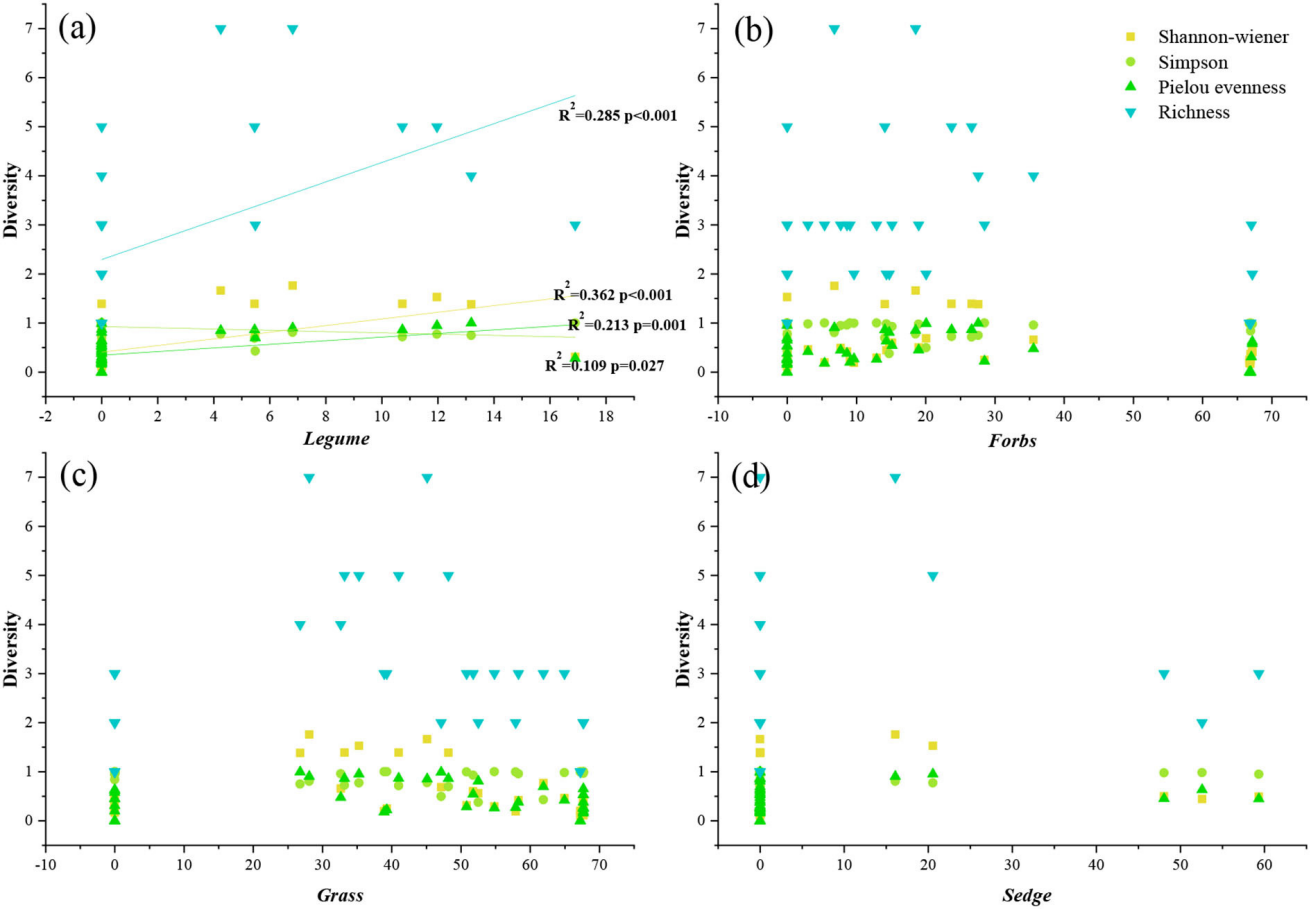
Relationships between PFGs and diversity. The solid line indicates a significant correlation (*p* < 0.05). The distribution of **(a–d)** represents the correlation analysis of legumes, forbs, grasses, sedges, and diversity indicators.

The results show that the diversity of plant communities at the same altitude was not consistent. The Shannon-wiener index was significantly higher than other sample points in A 1 and A 2, but the Simpson index was opposite to the Shannon-wiener index, and A 1 and A 2 were significantly lower than other sample points (Table 2). The Pielou Evenness index and Richness index changed more dramatically, but the indexes in A 1 and A 2 were also significantly higher than other sample points (Table 2). In the correlation analysis between diversity and PFGs, only legumes were significantly correlated with diversity indices (Figure 3). There was a significant positive correlation between Shannon-wiener, Pielou Evenness, Richness, and legume functional group, and a significant negative correlation between Simpson and legumes. But there was no significant correlation between other functional groups (forbs, grass, and sedge) and diversity indexes (Figure 3).

**Table 2:** Changes in different diversity indicators.

	**Shannon-wiener**	**Simpson**	**Pielou evenness**	**Richness**
A 1	1.09 ± 0.42^b^	0.61 ± 0.16^a^	0.91 ± 0.08^d^	3.60 ± 1.52^d^
A 2	1.42 ± 0.39^c^	0.70 ± 0.16^a^	0.85 ± 0.10^d^	5.40 ± 1.67^e^
A 3	0.28 ± 0.07^a^	0.95 ± 0.06^b^	0^a^	1.00^a^
A 4	0.31 ± 0.11^a^	0.98 ± 0.01^b^	0.24 ± 0.33^b^	1.40 ± 0.55^ab^
A 5	0.49 ± 0.07^a^	0.96 ± 0.02^b^	0.49 ± 0.10^c^	2.80 ± 0.45^cd^
A 6	0.25 ± 0.13^a^	0.99 ± 0.01^b^	0.31 ± 0.20^bc^	2.40 ± 0.55^bcd^
A 7	0.27 ± 0.15^a^	0.99 ± 0.01^b^	0.25 ± 0.24^b^	1.80 ± 0.84^abc^
A 8	0.30 ± 0.22^a^	0.99 ± 0.02^b^	0.34 ± 0.16^bc^	2.40 ± 0.89^cd^
A 9	0.28 ± 0.05^a^	1.00 ± 0.001^b^	0.28 ± 0.07^bc^	2.80 ± 0.45^bcd^

^a, b, c, d^Values with different superscripts are significantly different between and/or among the response variables within the column.

Within a column, significant differences between means (±SE, *n* = 5) are indicated by different letters (*post-hoc* Tukey's HSD test, *P* < 0.05).

### Correlation analysis between PFG soil plant C, N, and P

**Figure 4:**
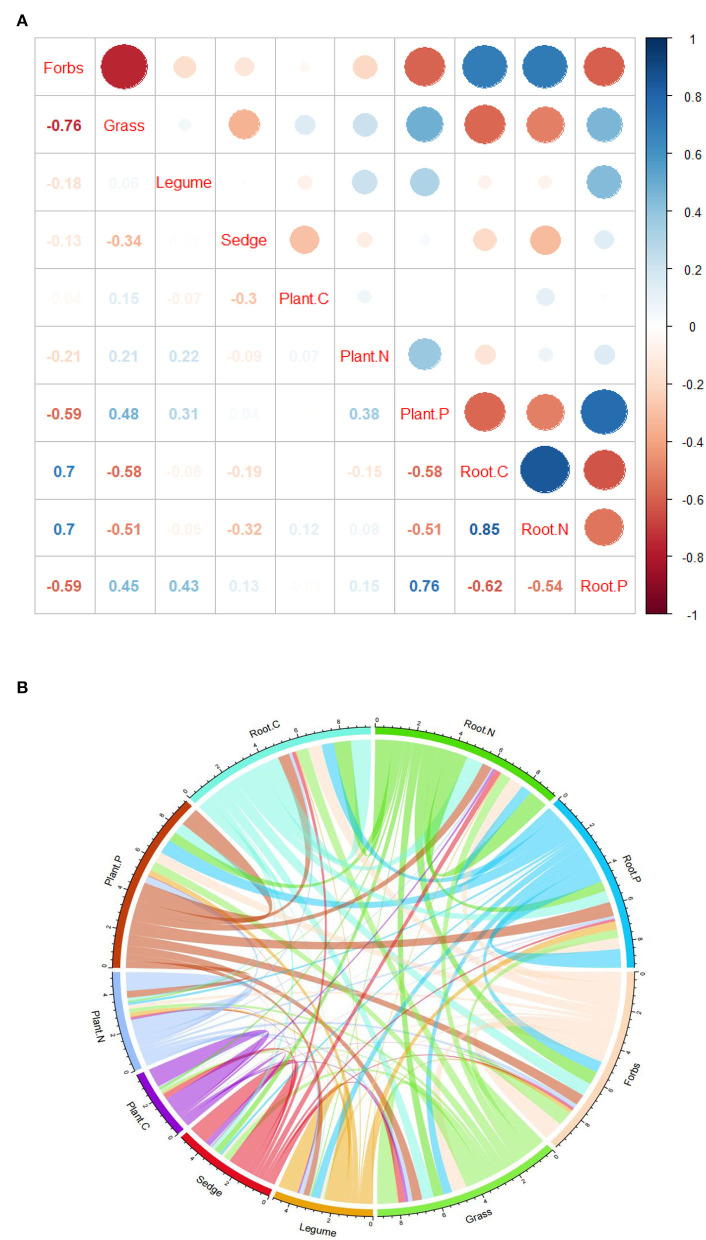
Correlations between PFGs with plant C, N, and P and root C, N, and P. **(A)** Red is negative, and blue is positive. The size of the circle represents the strength of the correlation. **(B)** The line thickness indicates the strength of the correlation.

The results show that there was a significant negative correlation between forbs and community plant P, and grasses, legumes, and community plant P showed a significant negative correlation. Sedge was positively correlated with community plant C (Figure 4A). But both forbs and grasses were significantly correlated with underground root nutrients. Forbs were positively correlated with C and N in underground root and negatively correlated with P in the underground root; forbs were negatively correlated with C and N in the underground root and positively correlated with P in the underground root; and legumes were only positively correlated with P in the underground root (Figure 4A). In addition, among the four functional groups in the study area, the sum of correlation coefficients of forb and grass was greater than that of legume and sedge and was more strongly correlated with C, N, and P of the plant community (Figure 4B). At the same time, forbs were negatively correlated with grasses. In the correlation analysis between PFGs and soil physical and chemical properties, the results showed different correlations with plant nutrients. Forbs were positively correlated with soil TP, and grasses were negatively correlated with soil TP (Table 3). In addition, legumes were positively correlated with soil SOC and soil TN; sedge was positively correlated with soil SOC and soil moisture (Table 3).

**Table 3:** Correlation analysis of PFGs and soil physical and chemical index.

	**Forbs**	**Grass**	**Legume**	**Sedge**
SOC	−0.202	−0.013	0.540**	0.409**
TN	−0.113	−0.054	0.545**	0.281
TP	0.600**	−0.586**	0.152	−0.216
pH	−0.183	0.214	−0.208	−0.084
Soil moisture	−0.205	0.010	−0.221	0.420**

The ^**^ indicates extremely significant correlation (*p* < 0.001).

### MLR analysis of functional groups and related influencing factors

**Figure 5:**
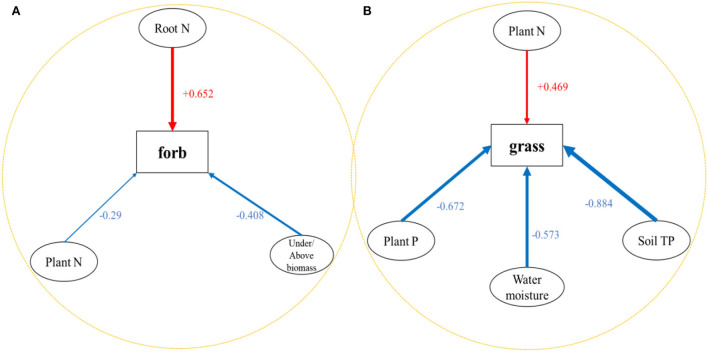
Schematic diagram of the relationship between **(A)** forb and **(B)** grass and different environmental factors. The blue arrow represents the negative effect, and the red arrow represents the positive effect.

We performed MLR on the main functional groups (forbs and grass) of all the sample sites. The results show that the forb was mainly affected by the under/above biomass (*p* < 0.001, *X*_1_), community plant N (*p* = 0.044, *X*_2_), and underground root N concentration (*p* = 0.018, *X*_3_). Under/above biomass and community plant N had negative effects on forbs, while underground root N had positive effects on forbs (Table 4A; Figure 5A). The MLR model of forbs is (1). Among them, underground root N contributed the most to forbs (T_17_ normalized coefficient is 0.652). The grass was mainly affected by soil TP (*p* = 0.012, *X*_1_), soil moisture (*p* = 0.022, *X*_2_), community TN (*p* = 0.011, *X*_3_), and community TP (*p* = 0.041, *X*_4_). The soil TP, water moisture, and community plant P had negative effects on grasses, while community plant N had positive effects on grasses (Table 4B; Figure 5B). The MLR model of grass is (2). Among them, soil TP contributed the most to grasses (T_10_ normalized coefficient is −0.884).


(1) $$\begin{array}{rc} Y & =-0.408X_{1}-0.29X_{2}+0.652X_{3} \end{array}$$

(2) $$\begin{array}{rc} Y & =-0.884X_{1}-0.573X_{2}+0.469X_{3}-0.672X_{4} \end{array}$$

**Table 4:** Statistical results of MLR parameters (*p* < 0.05).

	**Index**	**Nonstandardized coefficient**	**Normalized coefficient**	**T**	***p***	**VIF**
**(A)** ***Forbs***						
T	Constant	105.178		1.244	0.225	
T_1_	Shannon	18.694	0.329	0.738	0.467	27.532
T_2_	Simpson	35.461	0.215	1.072	0.294	5.571
T_3_	Pielou	−2.153	−0.027	−0.135	0.894	5.467
T_4_	Richness	−1.575	−0.091	−0.276	0.785	15.099
T_5_	Above-ground biomass	0.033	0.06	0.517	0.609	1.864
T_6_	Root biomass	0.153	0.06	0.413	0.683	2.918
T_7_	Under/above biomass	−6.04	−0.408	−4.082	0.000	1.393
T_8_	Soil SOC	−7.777	−0.573	−1.168	0.253	33.411
T_9_	Soil TN	33.193	0.271	0.556	0.583	32.968
T_10_	Soil TP	86.515	0.293	1.13	0.269	9.387
T_11_	pH	−13.247	−0.217	−1.423	0.167	3.22
T_12_	Soil moisture	1.36	0.291	1.551	0.133	4.904
T_13_	Plant C	−0.082	−0.15	−1.333	0.194	1.772
T_14_	Plant N	−1.767	−0.29	−2.119	0.044	2.598
T_15_	Plant P	21.231	0.382	1.53	0.138	8.676
T_16_	Root C	−0.042	−0.121	−0.435	0.667	10.742
T_17_	Root N	6.278	0.652	2.531	0.018	9.237
T_18_	Root P	−61.489	−0.315	−1.661	0.109	5.006
**(B)** ***Grass***
T	Constant	−58.171		−0.508	0.615	
T_1_	Shannon	16.928	0.276	0.494	0.625	27.532
T_2_	Simpson	−74.852	−0.419	−1.672	0.107	5.571
T_3_	Pielou	21.181	0.244	0.98	0.336	5.467
T_4_	Richness	−3.764	−0.201	−0.487	0.631	15.099
T_5_	Above-ground biomass	−0.086	−0.146	−1.008	0.323	1.864
T_6_	Root biomass	0.071	0.026	0.143	0.888	2.918
T_7_	Under/above biomass	0.052	0.003	0.026	0.979	1.393
T_8_	Soil SOC	−0.28	−0.019	−0.031	0.975	33.411
T_9_	Soil TN	−42.611	−0.322	−0.527	0.602	32.968
T_10_	Soil TP	−281.489	−0.884	−2.715	0.012	9.387
T_11_	pH	24.362	0.369	1.933	0.064	3.22
T_12_	Soil moisture	−2.89	−0.573	−2.434	0.022	4.904
T_13_	Plant C	0.103	0.176	1.243	0.225	1.772
T_14_	Plant N	3.087	0.469	2.735	0.011	2.598
T_15_	Plant P	−40.34	−0.672	−2.147	0.041	8.676
T_16_	Root C	0.114	0.307	0.88	0.387	10.742
T_17_	Root N	−5.598	−0.539	−1.668	0.107	9.237
T_18_	Root P	49.489	0.235	0.987	0.333	5.006

(A) DW = 2.262, (B) DW = 2.165.

## Discussion

In our study, PFGs responded differently to environmental changes and community competition. In this study area, the climate is dry and cold (Figure 1), so the dominant functional groups of the community are grasses and forbs (Figure 2). This is mainly because the forbs are the main dominant PFGs in the desert area and have strong adaptability to the arid environments (Ning et al., 2017). In contrast, grasses have conservative resource-use strategies and are strongly cold-tolerant, so they can adapt well to high-altitude environments (Suding et al., 2005). This is also consistent with the findings from other study areas of the central Tibetan Plateau at the same altitude, where the grassland vegetation types are dominated by grasses (Niu et al., 2019). At the same time, previous studies have shown that the growth of legumes will consume more water, and legumes could not survive in nutrient-poor desert areas, especially on mobile dunes (Guo et al., 2017; Cui et al., 2018). Therefore, in this study area, the dominant functional group of each sample site was forbs or grasses, and the importance value of both reached more than 50% of the community. The mass ratio hypothesis predicts that the species with the greatest proportion in the community will have the greatest impact on the ecological function of the community (Grime, 1998). In this study, forbs fit this hypothesis and have a significant correlation with community biomass; however, the grasses, legumes, and sedges did not fit the hypothesis, and the increase in grass functional groups only affected the root biomass of the community (Table 1). In species removal experiments in the arid northern prairies of Canada, removal of functional groups of grasses had the greatest impact on community biomass. However, the results indicate that the forbs had a dominant influence on the community in this area, which is inconsistent with previous studies that forbs have a dominant influence. This is mainly because the forbs are the main dominant PFGs in the desert area and have strong adaptability to the arid environments (Ning et al., 2017). Moreover, the grass functional group allocates less photosynthate to roots than to aboveground organs in alpine regions, serving as the main hindrance to efficient water uptake of the grasses (Wu Y. et al., 2013).

In addition to community biomass, PFGs are also related to community species diversity. Within the study area, species diversity, richness, and evenness of the community were significantly affected by legumes (Figure 3). The results indicate that the legume functional groups reflected the community change and development in the alpine grassland communities, with forbs and grasses as dominant functional groups (Hu et al., 2016). In addition, the dominant functional groups determined the community structure but could not reflect the development trend of the community. In other words, if a plant community is dominated by a few functional groups, then differences in species composition will have a greater impact on the whole system (Hooper and Vitousek, 1997). Therefore, the discovery of legume functional groups has a large effect on the prediction and discovery of ecosystem functioning in degraded grassland and extreme climate zones (Spehn et al., 2002; Scherer-Lorenzen et al., 2003). However, in previous studies in arid areas, legumes could not survive in nutrient-poor areas (Guo et al., 2017), so the changes in different PFGs in the community were related to nutrients.

Soil nutrients are the basis of plant growth, and the allocation of plant nutrients reflects the ecological strategies of plants to cope with environmental changes (Elser et al., 2010; Craine and Dybzinski, 2013). In this study area, forbs and grasses were the main components of plant communities, so the P concentration of plant communities and the C, N, and P concentrations of underground roots were mainly related to the functional groups of forbs and grasses (Figure 4). This is mainly because plants spend more resources on roots in less diverse and resource-poor environments, which is consistent with the alpine grassland ecological strategy of investing preferentially in structures for persistence (K-strategy) in previous studies (Patty et al., 2010; Ning et al., 2021). The legume functional group, as the key functional group affecting community diversity, was only significantly correlated with P in the community (Figure 4). Studies have shown that legumes are important species, affecting plant community diversity. These results indicate that P content was an important factor limiting grassland species diversity (Kattge et al., 2011). Nutrient limitation affects the composition of community functional groups and response strategies to environmental change. The study area is located at a high altitude of more than 3,700 m, where the climate is dry and the soil nutrient content is low. In all the communities we studied, grasses and forbs were significantly correlated with soil P concentration (Table 3), and as dominant functional groups, grasses and forbs had important effects on community composition. These results indicate that the soil P content was the main limiting factor affecting the plant community construction and the changes in main functional groups in this study area. This is consistent with previous studies that plant growth on the Qinghai-Tibet Plateau is mainly restricted by soil N and P, and the availability of N and P nutrients has an important effect on species composition and community structure (Janssens et al., 1998; Nie et al., 2010). The functional groups of legumes affected the contents of C and N in soil, and the changes in nitrogen fixation and community composition affected the absorption and transformation of C and N in soil (Hooper and Vitousek, 1998; Spehn et al., 2002). The results showed that the increase in the legume functional group not only affected the diversity of community species but also reduced the loss of soil C and N in the alpine desert grassland (Wen et al., 2013; Wu et al., 2018). The results indicate that different PFGs would adapt to the changes in the external environment according to their own traits under the same resource environment. In addition to soil nutrients, soil moisture is also one of the important factors affecting plant growth (Angers and Caron, 1998; Xu et al., 2018). However, in our study, only the sedge functional group and soil moisture had a significant correlation, while the major functional groups (forbs and grass) in the community showed no response to soil moisture (Table 3). The results indicate that functional groups such as grasses and forbs were less dependent on soil moisture than legumes and sedges (Cui et al., 2018). This is consistent with the previous studies that the increasing functional groups of forbs and grasses in arid areas can reduce the consumption of soil water and have strong adaptability to arid habitats (Wu et al., 2019). These studies suggest that the desert dominant PFGs can maintain a high utilization efficiency of soil nutrients under resource-limited conditions as a result of the function group's plasticity and ability to adapt to the extreme environment (Berendse and Aerts, 1987; Aerts and Chapin, 1999).

As an indicator of changes from species to the community level, PFGs are effective tools for biomass allocation, diversity change, and community composition (Wu J. S. et al., 2013; Bora et al., 2020). Changes in PFG tend not to be influenced by a single factor but by multiple factors, such as rainfall patterns, elevation gradients, nutrient availability, and atmospheric CO_2_ (Cramer et al., 2001; Weltzin et al., 2003). In this study, grasses and forbs, as dominant functional groups, were strongly responsive to a number of abiotic factors (Table 4; Figure 5). There was a strong direct correlation between grasses and forbs with community nutrients (N and P). In the analysis of the functional group of forbs, the aboveground community N concentration had a negative effect, while the underground root N concentration had a positive effect, but the results for grasses were the opposite of those for forbs (Table 4; Figure 5). This is mainly because most grasses have highly branched fibrous root systems than forb plants and can absorb nutrients in the surface soil more efficiently (Chapin et al., 1995; You et al., 2017). Therefore, the forbs allocate more nutrients to the root system to sustain growth, while the grasses allocate more nutrients to the growth of aboveground organs. This indicates that the plant community construction in this study area is mainly affected by soil N content, which is consistent with the current studies that the Qinghai-Tibet Plateau is mainly affected by N and P in previous studies (Nie et al., 2010). In addition, grasses had negative effects on soil moisture and soil TP while being affected by N content (Table 4B; Figure 5B). These results indicate that grasses have more competitive advantages over the fords due to their conservative resource-use strategies in extreme environments (Suding et al., 2005).

## Conclusion

In the alpine desert grassland, the species composition of PFGs is relatively single, but different PFGs play an important role in community composition and development. In our study, the grass and forb groups were the dominant functional groups in the study area, which had a great influence on the composition and structure of the community. The grass and forb groups adapted to environmental changes through the allocation of biomass and nutrients. However, the increase in legume groups can significantly improve the diversity and stability of the community. Due to the differences in the characteristics of the functional groups and their roles in the community, different PFGs had different responses to C, N, and P in soil and community. At the same time, the grass group had stronger adaptability than other PFGs under extreme environment. Overall, the adaptability of different PFGs to the environment is different. The composition and change in different PFGs can reflect the change and succession direction of a community.

## Data availability statement

The original contributions presented in the study are included in the article/Supplementary material, further inquiries can be directed to the corresponding author/s.

## Author contributions

AZ and XL jointly conceived and wrote the manuscript. FZ, YJ, and RW contributed to the experiments. All authors contributed to this study and approved the submitted version.

## Funding

This study was financially supported by the Second Tibetan Plateau Scientific Expedition and Research Program (2019QZKK0302), the National Natural Science Foundation of China (41877420), and the West Light Foundation of the Chinese Academy of Sciences (2019-FPGGRC).

## Conflict of interest

The authors declare that this study was conducted in the absence of any commercial or financial relationships that could be construed as a potential conflict of interest.

## Publisher's note

All claims expressed in this article are solely those of the authors and do not necessarily represent those of their affiliated organizations, or those of the publisher, the editors and the reviewers. Any product that may be evaluated in this article, or claim that may be made by its manufacturer, is not guaranteed or endorsed by the publisher.
