# Supplementary material for: Variation characteristics of different plant functional groups in alpine desert steppe of the Altun Mountains, northern Qinghai-Tibet Plateau
Source: Front Plant Sci. 2022 Sep 13;13:961692. doi: 10.3389/fpls.2022.961692 (PMC9513480; doi:10.3389/fpls.2022.961692)
Supplement: Supplementary file 1 [file Table_1.DOCX]

**Variation characteristics of different plant functional groups in alpine desert steppe of the Altun Mountains, northern Qinghai-Tibet Plateau**

**Ailin Zhang** **^a,b,c,d^, Xiangyi Li** **^a,b,c,d*^, Fanjiang Zeng ^a,b,c^, Yong Jiang ^e^, Ruzhen Wang ^e^**

^a^ State Key Laboratory of Desert and Oasis Ecology, Xinjiang Institute of Ecology and Geography, Chinese Academy of Sciences, Urumqi, 830011, China

^b^ Xinjiang Key Laboratory of Desert Plant Roots Ecology and Vegetation Restoration, Urumqi, 830011, China

^c^ Cele National Station of Observation and Research for Desert Grassland Ecosystems, Cele, 848300, Xinjiang, China

^d^ University of Chinese Academy of Sciences, Beijing, 100049, China

^e^ School of Life Sciences, Hebei University, Baoding 071002, China

* Corresponding authors.

*E-mail address*: [lixy@ms.xjb.ac.cn (Xiangyi](mailto:lixy@ms.xjb.ac.cn%20(Xiangyi) Li).

The following supplementary results are available for this manuscript:

**Table A1** Statistical table of sample species

| **Site** | **PFG** | **Species** |
| --- | --- | --- |
| A1 | Forbs | *Potentilla bifurca* Linn., *Krascheninnikovia compacta* (Losinsk.) Grubov, *Artemisia rutifolia*, *Sterigmostemum grandiflorum* |
|  | Grass | *Poa pratensis* L., *Stipa purpurea* Griseb. |
|  | Legume | *Astragalus arnoldii* Hemsl. |
|  | Sedge | *Carexenervis* C. A. Mey. subsp.enervis |
| A2 | Forbs | *Potentilla bifurca* Linn., *Krascheninnikovia compacta* (Losinsk.) Grubov |
|  | Grass | *Poa pratensis* L., *Stipa purpurea* Griseb. |
|  | Legume | *Leontopodium pusillum*, *Astragalus arnoldii* Hemsl. |
|  | Sedge |  |
| A3 | Forbs | *Krascheninnikovia compacta* (Losinsk.) Grubov, *Salsola abrotanoides* |
|  | Grass |  |
|  | Legume |  |
|  | Sedge |  |
| A4 | Forbs | *Salsola abrotanoides*, *Limonium aureum* (L.) Hill |
|  | Grass |  |
|  | Legume |  |
|  | Sedge |  |
| A5 | Forbs | *Glaux maritima* L., *Polygonum sibiricum* Laxm., *Triglochin maritimum* |
|  | Grass | *Calamagrostis pseudophragmites* (Haller f.) Koeler |
|  | Legume |  |
|  | Sedge | *Carexenervis* C. A. Mey. subsp.enervis |
| A6 | Forbs | *Salsola abrotanoides*, *Limonium aureum* (L.) Hill |
|  | Grass | *Leymus ovatus* (Trin.) Tzvel., *Stipa subsessiliflora* (Rupr.) Roshev. |
|  | Legume |  |
|  | Sedge |  |
| A7 | Forbs | *Krascheninnikovia compacta* (Losinsk.) Grubov, *Limonium aureum* (L.) Hill, *Salsola abrotanoides* |
|  | Grass | *Leymus ovatus* (Trin.) Tzvel., *Stipa subsessiliflora* (Rupr.) Roshev. |
|  | Legume | *Thermopsis alpina* (Pall.) Ledeb. |
|  | Sedge |  |
| A8 | Forbs | *Limonium aureum* (L.) Hill |
|  | Grass | *Leymus ovatus* (Trin.) Tzvel., *Stipa subsessiliflora* (Rupr.) Roshev. |
|  | Legume |  |
|  | Sedge |  |
| A9 | Forbs | *Artemisia rutifolia*, *Lepidium latifolium* Linnaeus |
|  | Grass | *Leymus ovatus* (Trin.) Tzvel., *Stipa subsessiliflora* (Rupr.) Roshev. |
|  | Legume |  |
|  | Sedge |  |
